# Moderate ethanol exposure reduces astrocyte-induced neuroinflammatorysignaling and cognitive decline in presymptomatic APP/PS1 mice

**DOI:** 10.21203/rs.3.rs-3627637/v1

**Published:** 2023-12-02

**Authors:** Shinwoo Kang, Jeyeon Lee, Sun Choi, Jarred Nesbitt, Paul H Min, Eugenia Trushina, Doo-Sup Choi

**Affiliations:** Mayo Clinic College of Medicine, and Science

**Keywords:** Alcohol use disorder, AUD, Alzheimer’s disease, AD, Moderate ethanol exposure, MEE, Apolipoprotein E, ApoE, low-density lipoprotein cholesterol, LDL-cholesterol

## Abstract

**Background:**

Alcohol use disorder (AUD) has been associated with the development of neurodegenerative diseases, including Alzheimer’s disease (AD). However, recent studies demonstrate that moderate alcohol consumption may be protective against dementia and cognitive decline.

**Methods:**

We examined astrocyte function, low-density lipoprotein (LDL) receptor-related protein 1 (LRP1), and the NF-κB p65 and IKK-α/β signaling pathways in modulating neuroinflammation and amyloid beta (Aβ) deposition. We assessed apolipoprotein E (ApoE) in the mouse brain using IHC and ELISA in response to moderate ethanol exposure (MEE). First, to confirm the intracerebral distribution of ApoE, we co-stained with GFAP, a marker for astrocytes that biosynthesize ApoE. We sought to investigate whether the ethanol-induced upregulation of LRP1 could potentially inhibit the activity of IL-1β and TNF-α induced IKK-α/β towards NF-κB p65, resulting in a reduction of pro-inflammatory cytokines. To evaluate the actual Aβ load in the brains of APP/PS1 mice, we performed with a specific antibody Aβ (Thioflavin S) on both air- and ethanol-exposed groups, subsequently analyzing Aβ levels. We also measured glucose uptake activity using 18F-FDG in APP/PS1 mice. Finally, we investigated whether MEE induced cognitive and memory changes using the Y maze, noble objective recognition (NOR) test, and Morris water maze (MWM).

**Results:**

Our findings demonstrate that MEE reduced astrocytic glial fibrillary acidic protein (GFAP) and ApoE levels in the cortex and hippocampus in presymptomatic APP/PS1 mice. Interestingly, increased LRP1 protein expression is accompanied by dampening the IKK-α/β-NF-κB p65 pathway, resulting in decreased IL-1β and TNF-α levels in male mice. Notably, female mice show reduced anti-inflammatory cytokines, IL-4, and IL-10 levels without altering IL-1β and TNF-α concentrations. In both males and females, Aβ plaques, a hallmark of AD, were reduced in the cortex and hippocampus of ethanol-exposed presymptomatic APP/PS1 mice. Consistently, MEE increased fluorodeoxyglucose (FDG)-positron emission tomography (PET)-based brain activities and normalized cognitive and memory deficits in the APP/PS1 mice.

**Conclusions:**

Our findings suggest that MEE may benefit AD pathology via modulating LRP1 expression, potentially reducing neuroinflammation and attenuating Aβ deposition. Our study implies that reduced astrocyte derived ApoE and LDL cholesterol levels are critical for attenuating AD pathology.

## Background

Alcohol use disorder (AUD) has been associated with the development of neurodegenerative diseases such as Alzheimer’s disease (AD) and Parkinson’s disease (PD) [1]. Furthermore, chronic and excessive alcohol consumption is a crucial risk factor for cognitive decline and alcohol-related dementia (ARD) [2, 3]. Recent studies have shown that excessive alcohol consumption reduces cognitive function in reversal learning [4, 5], indicating that excessive alcohol drinking dampens the ability to adapt to environmental changes [6]. On the other hand, it has been known that moderate alcohol consumption is possibly protective against cognitive decline [7–10]. However, it remained unclear how moderate ethanol exposure (MEE) prevents the accumulation of amyloid-beta (Aβ) peptides, reducing plaque formation in the brain [11] and protecting hippocampal neurons from Aβ toxicity [12].

APP/PS1 mice, a model of early-onset AD, have been used to study the molecular mechanisms of AD progression and therapeutic interventions [13–17]. We extensively characterized APP/PS1 mice, demonstrating that they recapitulate some aspects of human AD, including mitochondrial dysfunction, altered energy homeostasis [15], progressive accumulation of amyloid plaques [18], and cognitive dysfunction [14,16,17]. In addition, APP/PS1 mice are commonly used in studying ethanol-induced AD pathology [19, 20].

Cortical and hippocampal astrocyte reactivity is related to the development of AD [21]. Astrocytes synthesize ApoE and cholesterol, regulating cholesterol-dependent signaling in the brain [22]. In humans, the astrocytic ApoE4 allele, a genetic risk factor for AD, contributes to amyloidosis in neurons through increased ApoE4-derived cholesterol levels [23]. Moreover, astrocyte-derived cholesterol controls Aβ accumulation *in vivo,* linking ApoE, Aβ, and plaque formation, further underscoring astrocytes’ critical role in AD progression [24].

Cholesterol is known to regulate amyloid deposition in AD pathology [25,26]. Apolipoproteins play a crucial role in cholesterol transport and metabolism, and any changes in their levels can result in dysrégulation of lipid homeostasis in AD [27,28]. Astrocytes express several critical enzymes involved in cholesterol biosynthesis, such as 3-hydroxy-3-methyl glutaryl-CoA (HMG-CoA) reductase, and the uptake of these cholesterol-containing particles is dependent upon the low-density lipoprotein receptor (LDLR) in the plasma membrane [29]. Interestingly, LDLR-related protein (LRP) is involved in various cellular processes such as lipid metabolism, cell migration, and endocytosis [30]. Particularly, LRP1 activation inhibits Aβ aggregation [31,32] and down-regulates NF-κB-dependent proinflammatory cytokines [33,34], which are critical for AD pathology.

To determine a possible correlation between MEE and AD pathology, we examined how MEE alters cognitive function through LDL cholesterol, neuroinflammation, and Aβ deposition in age- and sex-dependent manner in APP/PS1 mice. Our data demonstrate the differential effect of MEE on AD pathology and suggest potential therapeutic targets.

## Materials and Methods

### Mice

All experimental procedures were approved by the Committee on Animal Care and Use at Mayo Clinic (A00005502-20). APP/PS1 mice harbor human mutations in APP_SWE_ (K670N, M671L) and PS1 (M146L) genes presenting the primary AD mechanisms, including Aβ deposition, mitochondrial dysfunction, inflammation, altered metabolic signatures, and energy homeostasis, and neurodegeneration [17]. In the presymptomatic group (12 ~ 24 weeks ethanol exposure), APP/PS1 mice and symptomatic group (36 ~ 48 weeks ethanol exposure) APP/PS1 mice were used in the study (Fig. S1A and B). The group size was determined based on similar studies conducted by our labs and others. All animals were housed individually in standard mouse cages under a 12-h artificial light-dark cycle with ad libitum access to food and water. Room temperature and humidity were kept constant (22 ± 1 °C; relative humidity: 55 ± 5%). Standard laboratory rodent chow (LabDiet 5P00 Prolab RMH 3000 rodent chow) and tap water were provided *ad libitum* throughout the experimental period. Mice underwent a battery of behavioral tests at baseline and various stages during ethanol exposure.

### Moderate Ethanol Exposure

As previously described [35], air or ethanol vapor was delivered in Plexiglas inhalation chambers (Fig. S2). We have previously used the vapor chamber system to successfully maintain stable blood ethanol concentration (BAC) in mice. Mice were exposed to ethanol vapor or room air using vapor administration chambers for 4 h from 09:00 to 13:00, followed by 20 h of room air in their home cages. This process was repeated for four consecutive days, followed by 3 days in their home cages with room air (withdrawal period). After the last ethanol exposure, the mice’s tail blood was collected immediately and centrifuged to extract the serum. BACs were measured by Analox GL5 multi metabolite analyzer (Analox Instruments, Stourbridge, United Kingdom) with the accompanying kits.

### FDG-PET

We performed micro-PET scanning using a Siemens Inveon MicroPET/CT Scanner (Siemens Preclinical Solutions Inc., Erlangen, Germany) with the 30 min list mode acquisition protocol. Mice were fasted one hour before the IP injection of 200 ~ 270 μCi of fludeoxyglucose F18(^18^F-FDG) in 200 μl injection volume prepared the same day at the Mayo Clinic Nuclear Medicine Animal Imaging Resource [36]. CT-based attenuation corrections were applied. During the scan, mice were anesthetized by inhalation of ~ 2% isoflurane supplemented with oxygen. PET images were spatially normalized to the mouse brain PET template [37] using PMOD v4.3 (PMOD technologies, Zurich, Switzerland). Then, brain ^18^F-FDG uptake was calculated as standard uptake value ratio (SUVR) with the cerebellum as referencing tissue. For group-wise comparisons, regional SUVRs were calculated as the average uptake over the total voxels in the region of interest (ROI).

### ELISA assay

The Aβ1-40,42, Apolipoprotein, HDL cholesterol, LDL cholesterol, and total cholesterol levels in the soluble fraction of frozen brain tissues were quantified using an ELISA assay (Control Aβ42, HDL cholesterol, LDL cholesterol, and total cholesterol were purchased from Thermo Fisher Scientific, Diego, CA, USA). All assays were performed according to manufacturer instructions. Levels of these proteins were calculated from a standard curve developed with specific optical density versus serial dilutions of a known concentration. Each standard and experimental sample was run in duplicate, and the results were averaged.

### Immunohistochemistry

For immunohistochemistry on frozen sections, the 35 μm-thick brain sections were washed three times in PBS containing 0.2% Triton X-100 and were then incubated in a blocking solution (0.5% bovine serum albumin and 3% normal goat serum in PBS with 0.4% Tween 20) for 1 h at RT21. The sections were incubated with the primary GFAP antibody (Novus Biologicals, Minneapolis, MN, USA) or Thioflavin S overnight at 4°C. Following this, the sections were washed three times and were then incubated with an Alexa Fluor 488 or Alexa Fluor 555 donkey anti-rabbit IgG antibody (Invitrogen, Carlsbad, CA, USA) for 1 h at RT. The brain slices were then washed three times and mounted onto slides using Antifade Mounting Medium with DAPI (Vector Laboratories, Burlingame, CA, USA). Tissue specimens were taken using a Nikon TS2-S-SM microscope equipped with a Nikon DS-Qi2 camera (Nikon Microscopy, Tokyo, Japan). Serial images of the cortex and hippocampus were captured on 4 consecutive 30 μm sections with x100 magnification. Once the ROIs were defined, we quantified the fluorescence intensity and percentage area of a red signal representing Alexa Fluro 555 within each ROI per section (5 mice per group)

### Y-maze spontaneous alternation

In the presymptomatic and symptomatic groups, mice were tested for spontaneous alternation in a three-armed Y-maze constructed from white polyvinyl plastic. Each arm (A, B, and C) was 40 cm long, 6.8 cm wide, and 15.5 cm high, and the three-folding angle was 120°. Mice were placed in the equipment and allowed to explore freely. During each 8-min period, the number of times that the tail of each animal fully entered each arm was counted for each arm, and then, the number of times each animal entered the arm one after another (in A, B, or C sequence) was also counted, which was assigned one point (actual alternation). The inside of the Y-maze was wiped with 70% ethanol between different animal trials. Alternation behavior was defined as no overlap into all three arms and was calculated by the following equation: Rate of spontaneous alterations (%) = (number of alternations) / (the total number of arm entries − 2) × 100. All tests were recorded by a technician blind to the genotype of the animals.

### Novel objective recognition task

We conducted a novel objective recognition (NOR) task with the young and symptomatic groups to assess the changes in cognition and memory. The setup comprised a black-walled square box measuring 35 × 35 × 30 cm. Mice were placed in the center of the open field box and allowed to acclimate for 30 min. Then, two of the same objects were placed in the box, and the rats were habituated for 10 minutes. After 1 hour, one object was replaced with a novel object, and the exploration time of the new or familiar object was recorded for 10 minutes using the Ethovision XT 9 system (Noldus Information Technology, Wageningen, Netherlands). The following parameters were measured: the total exploration time, frequency, objective recognition time, and memory index. The memory index is calculated by the exploration time for each object divided by the total exploration time.

### Open field test

The open field task is a simple sensorimotor test used to determine general activity levels, gross locomotor activity, and exploration habits in rodent models of CNS disorders. Presymptomatic and symptomatic mice were tested in a black-walled square box measuring 35 × 35 × 30 cm. The animal is placed in the arena and allowed to freely move about for 10 minutes while being recorded by an overhead camera. The footage is then analyzed by an automated tracking system for the following parameters: distance moved, velocity, and time spent in pre-defined zones.

### Morris water maze (MWM) test

Spatial learning and memory were assessed using the MWM test using the animal cognitive functions assessment meter (Ethovision Maze task system, Noldus Information Technology, Wageningen, Netherlands). Mice were tested in a circular pool (100 cm diameter, 45 cm high, and outer height 61.5 cm from the ground floor) filled with opaque water equilibrated to room temperature (22°C). The tank was divided into four quadrants with different navigation marks (cues) for each quadrant. Mice were continuously trained with 4 trials per mouse daily (once per navigation mark) for 4 days to search for the escape platform within a maximum of 60 s [38]. During the test, the platform location stayed constant, and the time taken to reach the platform was recorded as the escape latency. After the mouse found the hidden platform, it was kept on the platform for 2 s. If mice could not find the platform within 60 s, they were placed on the platform for 20 s to encode the location of the escape platform; for these trials, the escape latency was recorded as 60 s. Mice removed from the pool were dried and returned to the home cage. The MWM probe test was performed within 48 h of the final trial. The platform was removed from the pool, and the mice were placed in the water and allowed to swim for 60 s. The time spent in the quadrant that previously contained the platform indicates long-term memory maintenance. Swim distance, velocity, and frequency were recorded as measures of motor function. All the tests were performed by a technician who was blind to the genotype of the animals.

## Results

### Moderate ethanol exposure reduces ApoE and LDL cholesterol levels in presymptomatic APP/PS1

To examine the impact of ethanol exposure in the age- and sex-dependent early onset AD mouse model, we used two different age groups: presymptomatic group (ethanol exposure between 12 and 24 weeks of age, molecular and behavior experiments between 24–26 weeks of age) and symptomatic group (ethanol exposure between 36 and 48 weeks of age, molecular and behavior experiments between 48–50 weeks of age). Both male and female APP/PS1 mice were exposed to ethanol vapor or room air using vapor chambers for 4 h from 9 am to 1 pm, followed by 20 h of room air in their home cages to mimic alcohol drinking patterns in humans. This process was repeated for four consecutive days per week, followed by 3 days in their home cages with room air (withdrawal period) for 12 weeks to examine the long-term effect of alcohol exposure (Fig. S1A and B). After completing the 12-week cycle, we examined the effect of ethanol exposure as outlined in Fig. S1 A. We found no weight loss due to the influence of ethanol in both male and female presymptomatic or symptomatic APP/PS1 mice (Fig. S1C and D). The average blood alcohol concentration (BAC) immediately after the vapor chamber was approximately 170 mg/dl (Fig. SICand D).

**Figure 1:**
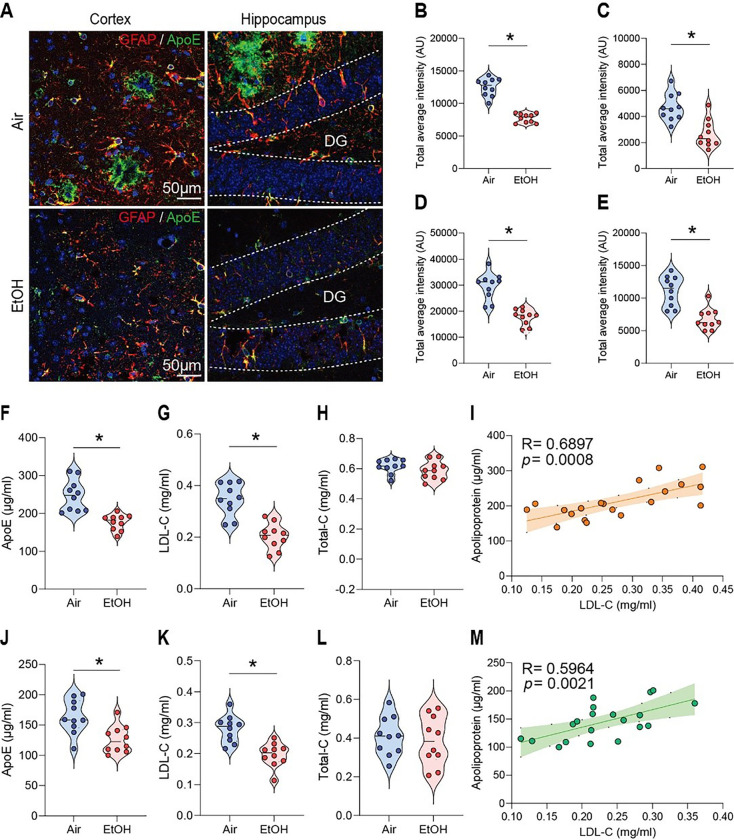
Impact of moderate ethanol exposure on ApoE and LDL cholesterol levels in the brains of presymptomatic APP/PS1 mice. (A) Representative immunohistochemistry images of ApoE (green) and GFAP (red) co-staining in the brains of air-exposed and ethanol-exposed APP/PS1 mice. (B and C) ApoE IHC evaluation revealed reduced ApoE levels in the cortex (B) and hippocampus (C) compared to the air-exposure group. (D and E) GFAP IHC evaluation showed decreased astrocyte activation in the cortex (D) and hippocampus (E) in the ethanol exposure compared to the air group. (F-H and J-L) Analysis of ApoE, LDL-cholesterol, and Total-cholesterol levels in the cortex and hippocampus by ELISA after moderate ethanol exposure. (F) ApoE level in the cortex, (G) LDL-cholesterol level in the cortex, (H) Total cholesterol level in the cortex, (I) Correlation in the cortex, (J) ApoE level in the hippocampus, (K) LDL-cholesterol level in the hippocampus, (L)Total cholesterol level in the hippocampus. (M)Correlation in the hippocampus. Data represent mean ± SEM; *n*= 10 per group. *P < 0.05 comparing each group. (B-l and K-N) Two-tailed Mann-Whitney test. (I and M) Spearman correlation analysis. Linear regression (solid line) and 95% confidence bands (shaded are) are shown. See Table S1 for full statistical information.

In AD, astrocytes surrounding amyloid plaques become reactive, indicating neuroinflammation, a key feature of AD pathology [24]. Since astrocytes express ApoE, which is critical for Aβ clearance [39], we first measured ApoE levels using immunohistochemistry (IHC) and enzyme-linked immunosorbent assay (ELISA) in response to MEE in presymptomatic APP/PS1 mice. To confirm the intracerebral distribution of ApoE, tissues were co-stained with GFARa marker for astrocytes that biosynthesize ApoE (Fig. 1A). Interestingly, we found a reduction of ApoE and astrocytes in the cortex (Fig. 1B and D) and hippocampus (Fig. 1C and E) of ethanol-exposed presymptomatic APP/PS1 mice compared to air-exposed counterparts. Consistently, we confirmed the reduced ApoE protein levels in ELISA experiments in the cortex (Fig. 1F) and hippocampus (Fig. 1J) of ethanol-exposed presymptomatic APP/PS1 mice. However, we found no sex-specific differences in GFAP and ApoE levels (Fig. S3A-C).

Since ApoE is an essential protein involved in cholesterol transport and late-onset AD [40,41], we examined whether MEE alters LDL-cholesterol levels in the brain. Interestingly, ethanol-exposed presymptomatic APP/PS1 mice have lower LDL-cholesterol levels than air-exposed counterparts (Fig. 1G, H, K, and L). However, the HDL-cholesterol levels did not differ between the air- and ethanol-exposed groups (Fig. S4A-H). A linear correlation of LDL cholesterol levels and ApoE (Fig. 1I and M) suggests that MEE reduces the biosynthesis of ApoE in astrocytes, which in turn causes a decrease in LDL cholesterol. On the other hand, we found no sex-specific difference (Fig. S5A-C).

### Increased LRP1 expression and function in presymptomatic APP/PS1 mice

**Figure 2:**
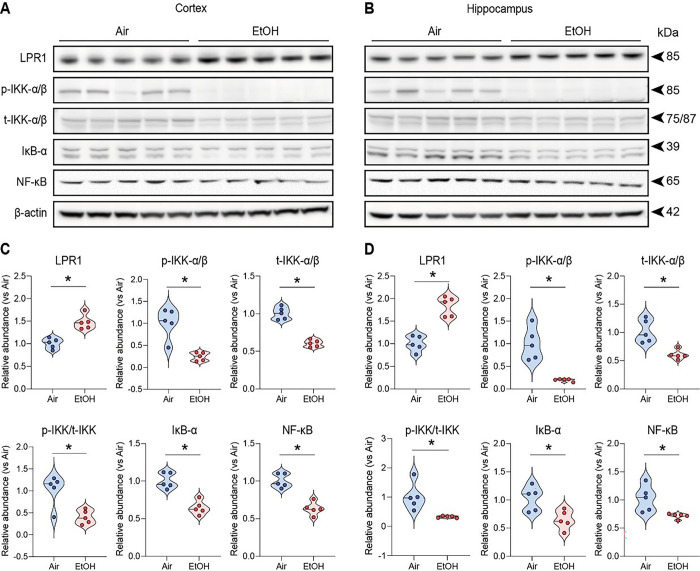
Effects of moderate ethanol exposure from IKK-α/β to kB-a on LRP1 expression and NF-κB signaling in presymptomatic APP/PS1 mice. (A and B) Western blot quantification of LRP1/IKK-α/β/kB-α/NF-κB signaling. (C and D) Western blots results of LRP1/IKK-α/β/kB-α/NF-κB in the cortex and hippocampus of the presymptomatic APP/PS1 mice brains. β-Actin was used as a loading control. (A and C) Cortex, (B and D) Hippocampus. Data represent mean ± SEM; *n* = 5 per group. *P < 0.05 comparing each group. Two-tailed Mann-Whitney test. See Fig. *S17* fora full Western blot and see Table S1 for full statistical information.

Since reduced ApoE and LDL cholesterol levels [42] are inversely associated with LRP1 expression, which regulates Aβ clearance in response to ApoE [43–45], we examined LDLR-related protein 1 (LRP1) levels between air-exposed and ethanol-exposed groups. Interestingly, we found a marked increase in LRP1 protein levels in the cortex (Fig. 2A and C) and hippocampus (Fig. 2B and D) of ethanol-exposed presymptomatic APP/PS1 mice compared to air-exposed counterparts. Consistent with previous findings, our results confirm the increase of LRP1 in response to reduced ApoE [46] or decreased LDL cholesterol levels [41].

### Moderate ethanol exposure reduces IL-1β &TNF-α induced by LRP1 –IKKα/β – NF-κB p65 pathway

**Figure 3:**
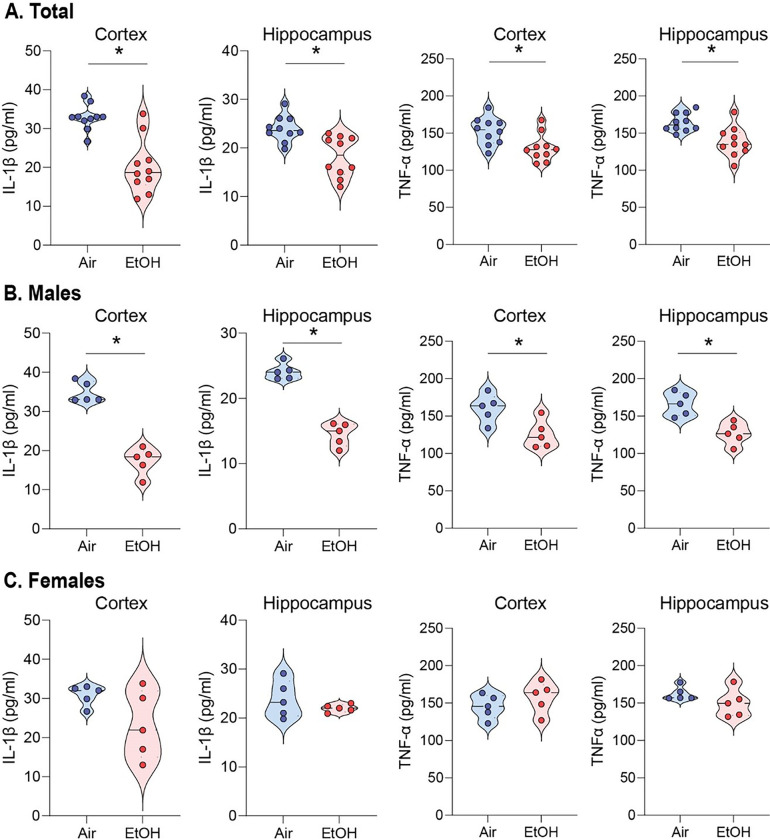
Moderate ethanol exposure significantly reduces IL-1β and TNF-α levels in both brain regions compared to air-exposed presymptomatic APP/PS1 mice. (A-C) ELISA assay to detect IL-1β and TNF-α levels in the cortex and hippocampus of presymptomatic APP/PS1 mice. (A) Comparison of air and ethanol groups including male and female (B) Comparison of only male mice air and ethanol groups (C) Comparison of only female mice air and ethanol groups. Data represent mean ± SEM; *n*= 5 per group. *P < 0.05 comparing each group. Two-tailed Mann-Whitney test. See Table S1 for full statistical information.

Next, we investigated whether the ethanol-induced upregulation of LRP1 reduces proinflammatory cytokines since activating LRP1 is known to down-regulate NF-κB-dependent cytokine expressions [31, 61]. First, we examined ten different cytokines and found that IL-1β and TNF-α were reduced in the serum of ethanol-exposed presymptomatic males but not in female APP/PS1 mice (Fig. S6A-J). IL-1β and TNF-α are cytokines that may promote inflammation by activating the transcription factor NF-κB p65 [47]. Remarkably, we observed a reduction in p-IKK-α/β levels in ethanol-exposed presymptomatic APP/PS1 mice compared to air-exposed counterparts (Fig. 2A–D). This decrease in p-IKK-α/β led to a corresponding reduction in NF-κB p65 protein levels (Fig. 2A–D). We further assessed the IL-1β and TNF-α levels in the cortex and hippocampus using ELISA (Fig. 3). After 12 weeks of ethanol exposure, as expected, we found reduced levels of IL-1β and TNF-α in the cortex and hippocampus of ethanol-exposed presymptomatic APP/PS1 mice (Fig. 3A). Surprisingly, we found that IL-1β and TNF-α levels were reduced only in male but not in female mice (Fig. 3B and C). Instead, we found an upregulation of IL-10, an anti-inflammatory cytokine, in the hippocampus of ethanol-exposed presymptomatic APP/PS1 female mice (Fig. S7A-D).

### Moderate ethanol exposure reduced A0 plaques in presymptomatic APP/PS1 mice

**Figure 4:**
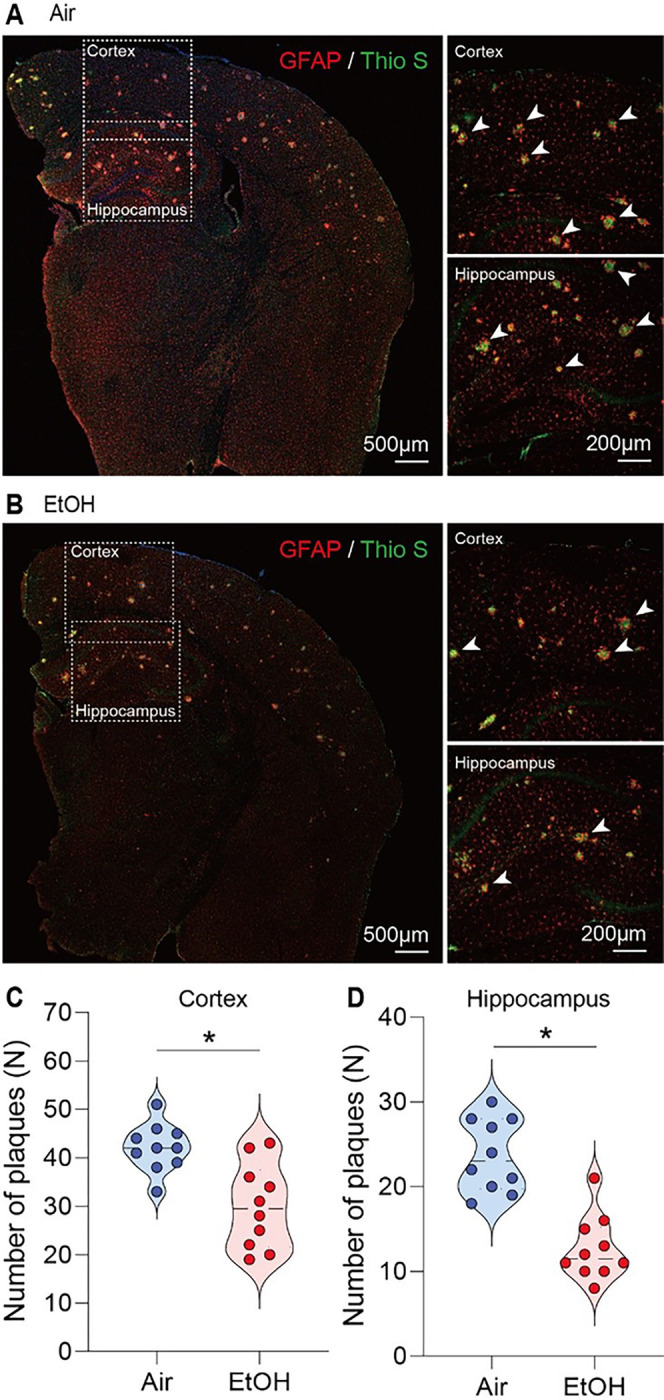
Reduction of Aβ plaque formation in the cortex and hippocampus of ethanol-exposed presymptomatic APP/PS1 mice. Immunohistochemistry analysis shows the effects of MEE on Aβ plaque formation in the cortex and hippocampus of presymptomatic APP/PS1 mice. (A and B) Aβ plaques were visualized using Thioflavin S staining. (C and D) the number of plaques was significantly decreased in both cortex and hippocampus of ethanol-exposed presymptomatic APP/PS1 mice compared to air-exposed controls. Data represent mean ± SEM; *n* = 10 per group. *P < 0.05 comparing each group. Two-tailed Mann-Whitney test. See Table S1 for full statistical information.

Then, we questioned whether MEE could potentially mitigate the deposition of Aβ plaques [48,49]. We utilized a simple and straightforward thioflavin S staining to evaluate the Aβ load in the brains of presymptomatic APP/PS1 mice [38, 50]. We quantified the number of Aβ plaques, including intra-neuronal Aβ and extracellular plaques. We compared these in the cortex and dentate gyrus of the hippocampus across all experimental presymptomatic APP/PS1 mice (Fig. 4A–B). Our results revealed a notable reduction in Aβ plaques in the cortex (Fig. 4C) and hippocampus (Fig. 4D) in both males and females (Fig. S8 A-C) in response to ethanol exposure in presymptomatic APP/PS1 mice compared to air-exposed counterparts.

**Figure 5:**
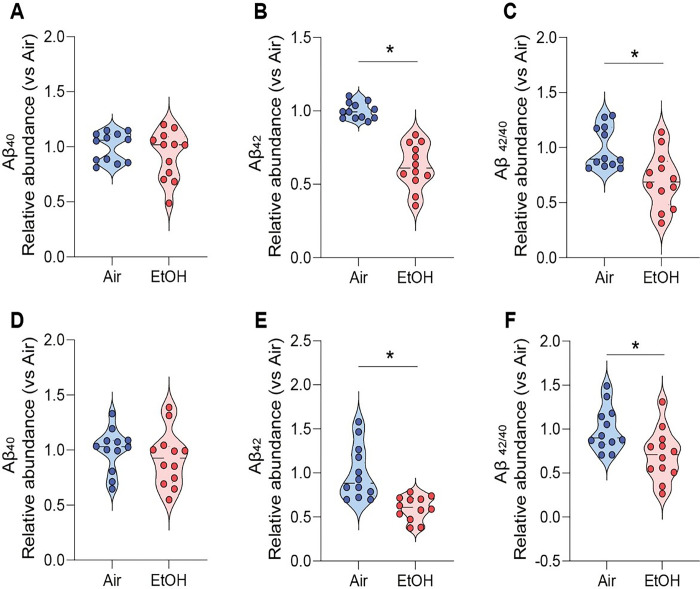
Reduction of Aβ levels in cortex and hippocampus of ethanol-exposed presymptomatic APP/PS1 mice. ELISA analysis shows the effects of chronic ethanol exposure on Aβ levels in the cortex and hippocampus of presymptomatic APP/PS1 mice. Quantification of amyloid Aβ1-40,42 levels using ELISA revealed a significant reduction in the cortex (A-C) and hippocampus (D-F) of ethanol-exposed presymptomatic APP/PS1 mice after 12 weeks of ethanol exposure compared to age-matched air-exposed presymptomatic APP/PS1 mice. Data represent mean ± SEM; *n* = 12 per group. *P < 0.05 comparing each group. Two-tailed Mann-Whitney test. See Table S1 for full statistical information.

We next performed an ELISA assay to detect Ap_42_ in brain tissue supernatants collected from both experimental groups to quantify the level of amyloid protein in the brain. At 12 weeks of ethanol exposure, Aβ_42_ levels were markedly reduced in the cortex and hippocampus of ethanol-exposed presymptomatic APP/PS1 mice compared to air-exposed presymptomatic APP/PS1 mice (Fig. 5A–F). Interestingly, we found no sex-specific differences in Aβ plaques (Fig. S8 A-C), indicating that two different neuroinflammatory signaling pathways yield similar effects on the Aβ plaques in presymptomatic APP/PS1 mice. In addition, we observed no notable differences in Aβ levels in ethanol-exposed symptomatic APP/PS1 mice (Fig. S9 A-D).

### Increased brain metabolism after ethanol exposure in presymptomatic APP/PS1 mice

**Figure 6:**
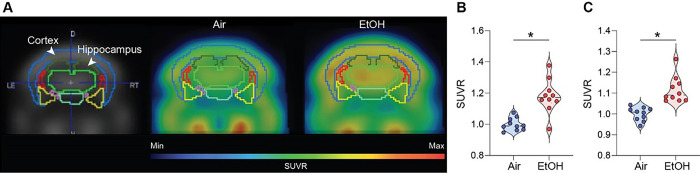
[^18^F] Fluorodeoxyglucose uptake measured by in vivo microPET. (A) Representative FDG-PET images from air-exposed and ethanol-exposed presymptomatic APP/PS1 mice. The boundaries of ROIs were drawn on the coronal section. Glucose uptake increased in the cortex and hippocampus of ethanol-exposed presymptomatic APP/PS1 mice. Quantification of glucose uptake by FDG-PET imaging in the cortex (B) and hippocampus (C). Data represent mean ± SEM; *n* = 10 per group. *P < 0.05 comparing each group. Two-tailed Mann-Whitney test. See Table S1 for full statistical information.

The fluorodeoxyglucose-positron emission tomography (FDG-PET) measures glucose metabolism and brain activity [51]. Thus, we performed the micro-PET experiments using the ^18^F-FDG radiotracer to examine whether MEE alters brain activity. We noted a higher uptake of ^18^F-FDG in the ethanol-exposed presymptomatic APP/PS1 mice compared to the air-exposed presymptomatic APP/PS1 mice, especially in the cortex and hippocampus (Fig. 6A–C) without sex-specific differences (Fig. S10A). However, our results show no difference in FDG uptake between the groups in symptomatic APP/PS1 mice (Fig. S11A-C), indicating that MEE increases brain activity in presymptomatic APP/PS1 mice without apparent impact on symptomatic APP/PS1 mice.

### Moderate ethanol exposure restores cognitive, memory, and reversal learning deficits in presymptomatic APP/PS1 mice

**Figure 7:**
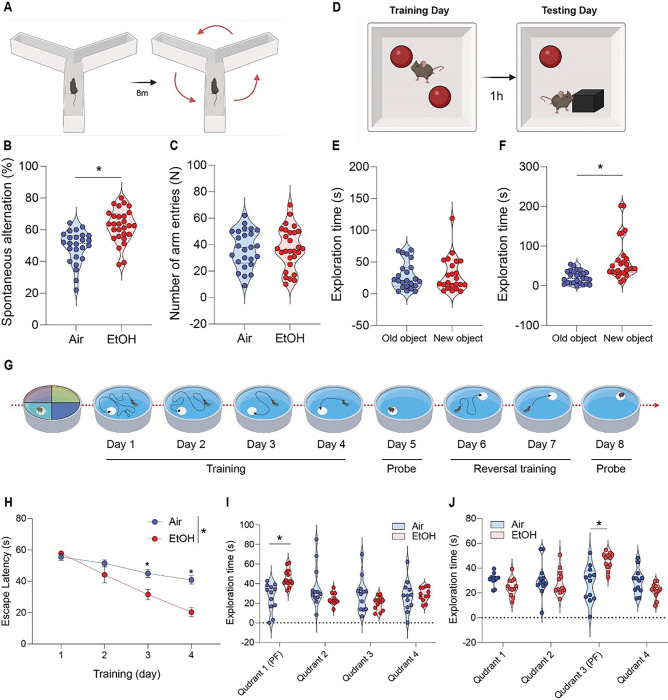
Effect of moderate ethanol exposure on cognitive performance in presymptomatic APP/PS1 mice. (A) Y-maze schematic diagram, (B) Y-maze test showing spontaneous alteration rate and (C) total arm entries for air-exposed and ethanol-exposed presymptomaticAPP/PS1 mice. (D) Novel Object Recognition (NOR) task schematic diagram, (E and F) NOR task demonstrating time spent exploring familiar and unfamiliar objects for both groups. (G) Morris water maze (MWM) schematic diagram, (H and I) MWM acquisitiontraining and probe test (J) reversal probe test. Data represent mean ± SEM; *n*= 12~28 per group. *P < 0.05 comparing each group. (B-C and E-F) Two-tailed Mann-Whitney test (H and J) Two-way ANOVA followed by Tukey’s multiple comparisons tests. See Table S1 for full statistical information.

Finally, we investigated whether MEE could rescue cognitive and memory functions in presymptomatic APP/PS1 mice. Our primary analysis involved evaluating the impact of ethanol exposure on spatial and procedural working memories in the Y-maze (Fig. 7A). Notably, we found an enhanced spontaneous alteration in the ethanol-exposed presymptomatic APP/PS1 compared to the air-exposed presymptomatic APP/PS1 mice (Fig. 7B) without changes in a total number of arm entries (Fig. 7C). Next, in novel object recognition (NOR) task (Fig. 7D), the ethanol-exposed presymptomatic APP/PS1 mice exhibited a prolonged exploration time of the unfamiliar object (Fig. 7E). In contrast, air-exposed presymptomatic APP/PS1 mice displayed similar exploration times for both familiar and unfamiliar objects (Fig. 7F) without differences in total exploration time, speed, total distance, and frequency (Fig. S12A-D). Importantly, as reported [52–54], we noted that presymptomatic APP/PS1 mice exhibited cognitive and memory decline in Y-maze and NOR compared to aged-matched non-transgenic (NTG) mice. Interestingly, we found that MEE has no effect on cognitive performance in presymptomatic NTG mice (Fig. S13A-D). Also, symptomatic APP/PS1 mice exhibited no difference in cognitive and memory function in response to MEE (Fig. S14A-K). Our findings indicate that MEE rescues or normalizes cognitive function in presymptomatic APP/PS1 mice without affecting presymptomatic NTG or symptomatic APP/PS1 mice.

We next tested spatial reference and working memory in the Morris water maze (MWM) (Fig. 7G). As previously published [38], we measured spatial learning on the fourth day during the acquisition training. Consistently with Y-maze and NOR, the ethanol-exposed presymptomatic APP/PS1 mice showed a shorter latency to find a safe platform than the air-exposed counterparts (Fig. 7H). In a subsequent probe test, ethanol-exposed presymptomatic APP/PS1 mice exhibited an increased preference for the target quadrant relative to other quadrants (Fig. 7I). At the same time, the swimming distance remained similar between groups (Fig. S12E and F). We next assessed the reversal learning. Presymptomatic APP/PS1 mice showed enhanced cognitive function, indicating that the ethanol-exposed presymptomatic APP/PS1 mice are more flexibly adapted to the altered environment (Fig. 7J). We conducted an open-field test to ensure whether mice’s locomotor activity affects memory and cognitive abilities. We found no differences between the air- and ethanol-exposed groups regarding distance moved or velocity (Fig. S12G and FI). These results demonstrate that MEE only restores cognitive and memory function in alcohol-exposed male and female presymptomatic APP/PS1 mice (Fig. S15 A-H).

## Discussion

This study provides a novel insight into how MEE contributes to cognitive improvement in the early-onset AD model, the APP/PS1 mice [19, 55]. Our findings indicate that an MEE reduces astrocyte activity and ApoE biosynthesis, which may lower the brain’s low-density lipoprotein (LDL) cholesterol levels. Also, consistent with a previously reported inverse correlation between ApoE and LRP1 expression [56], we observed increased LRP1 protein levels in the brains of ethanol-exposed presymptomatic APP/PS1 mice.

It is worth noting that our initial hypothesis was that a short (binge-like) and naturalistic ethanol exposure might exacerbate AD pathology. However, our findings contradict our initial hypothesis. Although excessive drinking often refers to more than 4 drinks per day for women and more than 5 drinks per day for men [57], moderate alcohol consumption has been reported to provide some health benefits, especially cardiovascular health [58]. Keeping these in mind (4–6), it is still an open question how to define “moderate” or “non-hazardous” drinking. In our study, contrasting to our previous [35] or other studies [59], we chose to expose mice to vaporized alcohol for 4 h per day without using pyrazole, an inhibitor of alcohol dehydrogenase (ADH), which naturally and slowly increases BAC to approximately 170 mg/dl. Although 170 mg/dl is not typically a moderate dose of alcohol, we consider this as a moderate dose because of relatively short alcohol exposure and ten times faster heart rate in mice compared to humans [60]. As we reported [35], longer (16 h per day, 4 days per week, 4 weeks) daily ethanol exposures in the vapor chamber may worsen the AD-like pathology in APP/PS1 mice.

In the presymptomatic group, MEE reduced Aβ plaque count and levels, with a corresponding improvement in cognitive function. This suggests that moderate alcohol consumption could mitigate neuronal loss and improve cognitive function in early disease stages. Importantly, we found that MEE has no effect on presymptomatic NTG mice nor symptomatic APP/PS1 mice (Fig. S13 and Fig. S14), suggesting that MEE normalizes AD-like cognitive function in only presymptomatic APP/PS1 mice. Our results imply that possible neuroprotective effects of moderate alcohol consumption may not extend to the symptomatic group, who already have an established Aβ plaque burden and more advanced neurodegeneration [61].

In addition to the addictive nature of alcohol [62], alcohol is known to damage multiple organs and cause many diseases, including cancers [63]. In particular, prolonged alcohol misuse causes alcohol liver disease [64] and hepatitis [65]. The AST and ALT levels are hallmarks of liver function [66]. We found increased AST levels without altered ALT levels in presymptomatic APP/PS1 mice liver compared to aged-matched air-exposed APP/PS1 mice without differences in the cortex and hippocampus (Fig. S16A-C). The AST/ALT ratio of over 1.5 is considered severe liver damage [67, 68]. In this regard, a relatively high AST/ALT ratio (~ 2.3) in the liver of APP/PS1 mice indicates that the ethanol exposure paradigm has a detrimental effect on liver function (Fig. S16A-C). Thus, despite some beneficial effects of alcohol in presymptomatic APP/PS1 mice, because only about 10% are early-onset AD patients and a few of them have APP/PS1 mutations [69–71], our findings do not support alcohol drinking to prevent cognitive decline or AD pathology. Instead, we want to emphasize that even moderate drinking could be harmful for those especially sensitive to the intoxication effects of alcohol.

Despite the unclear mechanistic causality of altered LRP1 expression, our study establishes a compelling correlation between MEE and changes in ApoE, LDL cholesterol, and LRP1 levels. Additionally, our study revealed that ethanol exposure significantly mitigates the levels of proinflammatory cytokines, IL-1β and TNF-α, in the cortex and hippocampus of ethanol-exposed APP/PS1 mice compared to air-exposed counterparts, which is causally linked to the inhibition of the LRP1-IKK-α/β-NF-κB p65 pathway. This finding is particularly significant as both LRP1 and Toll-like receptor 4 (TLR4) play influential roles in neuroinflammation and AD pathogenesis [72,73]. LRP1 suppresses microglial and astrocytic cell activation, critical contributors to neuroinflammation, by regulating the TLR4/NF-κB p65/MAPK signaling pathway [72]. This interaction regulates the release of proinflammatory cytokines and phagocytosis, contributing to maintaining brain homeostasis [74]. Flowever, IκB-α levels responded to ethanol within 30 minutes in mixed hippocampal cell samples from wild-type mice but not in cells from TLR4- or MyD88-deficient mice [75]. Besides, insufficient LRP1 activation is associated with inflammation-induced tumor progression [76,77], demonstrating the role of LRP1 in inflammation. Our findings illustrate alterations in sub-signals IKK-α/β and IKβ-α, notwithstanding the absence of an overall alteration in TLR4. Interestingly, a peptide ligand SP16 is known to activate LRP1, which decreases inflammation and increases cell survival in acute myocardial infarction [78,79]. A recent phase 1 clinical trial as a first-in-class anti-inflammatory LRP1 agonist shows a promising outcome in healthy volunteers [80]. Moreover, a new peptide agonist, COG1410, shows a similar anti-inflammatory effect in rats [61]. Thus, a future study will reveal a possible therapeutic effect of LRP1 agonist in neuroinflammation-related AD.

One intriguing finding is the reduced levels of proinflammatory cytokine IL-1β in male mice following MEE. IL-1β plays a significant role in inducing neuroinflammation, and its reduction suggests a decrease in the inflammatory response [81]. This reduction could potentially lead to alleviating the symptoms associated with neuroinflammation, such as cognitive dysfunction. Conversely, in female mice, MEE has been linked to the upregulation of IL-10, an anti-inflammatory cytokine. An increase in IL-10 suggests an enhanced anti-inflammatory response, potentially protecting the brain from the damaging effects of inflammation [81, 82]. This upregulation might contribute to preserving cognitive function and neuronal health in female mice. Interestingly, we found no sex-specific changes in AD-like behaviors but only sex-specific changes in cytokine levels (Table 1). Although the exact mechanisms of these sex-specific effects of ethanol are unclear, several plausible explanations underlie our findings. Hormonal differences between males and females, such as the influence of estrogen and testosterone, could potentially differentially respond to ethanol exposure [83]. Several recent studies revealed the sex-specific differences in the regulation of cytokines by astrocytes and microglia. In males, astrocytes primarily regulate proinflammatory cytokines [84]. Conversely, in females, microglia play a central role in regulating anti-inflammatory cytokines [85]. Furthermore, astrocytes are more active in anti-inflammatory phenotype in females [86]. These differences underscore the complex interplay of astrocytes, microglia, and cytokines and their roles in the brain. Similarly, recent studies have shown that astrocytes and microglia, essential central nervous system (CNS) components, respond differently to alcohol exposure. Astrocytes primarily activate proinflammatory cytokines, proteins that heighten inflammation in response to alcohol consumption. This is part of their role in maintaining CNS homeostasis and their varying response to inflammatory stimuli [82, 87]. On the other hand, microglia mainly activate anti-inflammatory cytokines, undermining the inflammation. Focusing on the role of TLR4 in alcohol-induced neuroinflammation and brain damage, alcohol consumption activates microglia through TLR4to produce anti-inflammatory cytokines in the brain [88]. Thus, our findings imply that MEE preferentially reduces astrocyte-induced proinflammatory cytokines in males while increasing microglia-induced anti-proinflammatory cytokines; both yield similar MEE-induced behavior outcomes. Further research may reveal mechanisms underlying the sex difference in cytokine-mediated signaling and AD pathology.

## Conclusion

Our study cogently demonstrates that reducing neuroinflammation and LDL cholesterol would have a therapeutic effect on cognitive impairment in AD, evidenced by the rescue of cognitive and memory deficits in the presymptomatic APP/PS1 mice. Considering the harmful effect on liver function and addiction liability of alcohol, our findings do not support even moderate drinking. However, our findings offer a new insight that activation of LRP1 could be a therapeutic target for mitigating neuroinflammation and attenuating Aβ deposition, thereby ameliorating AD pathology.

## Figures and Tables

**Table 1:** Summary of statistical analysis

Figure		Statistical Tests	Comparison	Value	P value
Figure 1	**B**	Mann-Whitney test	**Air vs EtOH**	U = 0	P < 0.0001
	**C**	Mann-Whitney test	**Air vs EtOH**	U = 9	P = 0.0011
	**D**	Mann-Whitney test	**Air vs EtOH**	U = 1	P < 0.0001
	**E**	Mann-Whitney test	**Air vs EtOH**	U = 4	P = 0.0001
	**F**	Mann-Whitney test	**Air vs EtOH**	U = 1.5	P < 0.0001
	**G**	Mann-Whitney test	**Air vs EtOH**	U = 4	P = 0.0001
	**H**	Mann-Whitney test	**Air vs EtOH**	U = 37	P = 0.3423
	**I**	Correlation	**ApolipoproteinE vs LDL-C**	R = 0.6897	P = 0.0008
	**J**	Mann-Whitney test	**Air vs EtOH**	U = 13	P = 0.0039
	**K**	Mann-Whitney test	**Air vs EtOH**	U = 18	P = 0.0138
	**L**	Mann-Whitney test	**Air vs EtOH**	U = 47	P = 0.8534
	**M**	Correlation	**ApolipoproteinE vs LDL-C**	R = 0.5964	P = 0.0021
Figure 2	**C (LRP1)**	Mann-Whitney test	**Air vs EtOH**	U = 0	P = 0.0079
	**C (p-IKK-α/β)**	Mann-Whitney test	**Air vs EtOH**	U = 0	P = 0.0079
	**C (t-IKK-α/β)**	Mann-Whitney test	**Air vs EtOH**	U = 0	P = 0.0079
	**C (p-/t-IKK-α/β)**	Mann-Whitney test	**Air vs EtOH**	U = 2	P = 0.0317
	**C (IκB-α)**	Mann-Whitney test	**Air vs EtOH**	U = 0	P = 0.0079
	**C** (NF-κB)	Mann-Whitney test	**Air vs EtOH**	U = 0	P = 0.0079
	**D (LRP1)**	Mann-Whitney test	**Air vs EtOH**	U = 0	P = 0.0079
	**D (p-IKK-α/β)**	Mann-Whitney test	**Air vs EtOH**	U = 0	P = 0.0079
	**D (t-IKK-α/β)**	Mann-Whitney test	**Air vs EtOH**	U = 0	P = 0.0079
	**D (p-/t-IKK-α/β)**	Mann-Whitney test	**Air vs EtOH**	U = 0	P = 0.0079
	**D (IκB-α)**	Mann-Whitney test	**Air vs EtOH**	U = 2	P = 0.0317
	**D** (NF-κB)	Mann-Whitney test	**Air vs EtOH**	U = 0	P = 0.0079
Figure 3	**A (Total, IL-1β, Cortex)**	Mann-Whitney test	**Air vs EtOH**	U = 10	P = 0.0014
	**A (Total, IL-1β, Hippocampus)**	Mann-Whitney test	**Air vs EtOH**	U = 9.5	P = 0.0011
	**A (Total, TNF-α, Cortex)**	Mann-Whitney test	**Air vs EtOH**	U = 18	P = 0.0147
	**A (Total, TNF-α Hippocampus)**	Mann-Whitney test	**Air vs EtOH**	U = 12	P = 0.0018
	**B (Male, IL-1β, Cortex)**	Mann-Whitney test	**Air vs EtOH**	U = 0	P = 0.0079
	**B (Male, IL-1β, Hippocampus)**	Mann-Whitney test	**Air vs EtOH**	U = 0	P = 0.0079
	**B (Male, TNF-α Cortex)**	Mann-Whitney test	**Air vs EtOH**	U = 2	P = 0.317
	**B (Male, TNF-α Hippocampus)**	Mann-Whitney test	**Air vs EtOH**	U = 0	P = 0.0079
	**C (Female, IL-1β, Cortex)**	Mann-Whitney test	**Air vs EtOH**	U = 7	P = 0.3905
	**C (Female, IL-1β, Hippocampus)**	Mann-Whitney test	**Air vs EtOH**	U = 9	P = 0.5476
	**C (Female, TNF-α Cortex)**	Mann-Whitney test	**Air vs EtOH**	U = 6	P = 0.2222
	**C (Female, TNF-α Hippocampus)**	Mann-Whitney test	**Air vs EtOH**	U = 5	P = 0.1508
Figure 4	**B**	Mann-Whitney test	**Air vs EtOH**	U = 13	P = 0.0038
	**C**	Mann-Whitney test	**Air vs EtOH**	U = 3.5	P < 0.0001
Figure 5	**A**	Mann-Whitney test	**Air vs EtOH**	U = 56	P = 0.3698
	**B**	Mann-Whitney test	**Air vs EtOH**	U = 0	P < 0.0001
	**C**	Mann-Whitney test	**Air vs EtOH**	U = 23	P = 0.0036
	**D**	Mann-Whitney test	**Air vs EtOH**	U = 50	P = 0.2133
	**E**	Mann-Whitney test	**Air vs EtOH**	U = 10	P = 0.0001
	**F**	Mann-Whitney test	**Air vs EtOH**	U = 29	P = 0.0121
Figure 6	**B**	Mann-Whitney test	**Air vs EtOH**	U = 7	P = 0.0005
	**C**	Mann-Whitney test	**Air vs EtOH**	U = 0	P < 0.0001
Figure 7	**B**	Mann-Whitney test	**Air vs EtOH**	U = 116.5	P < 0.0001
	**C**	Mann-Whitney test	**Air vs EtOH**	U = 359.5	P = 0.9623
	**E (Air)**	Mann-Whitney test	**Air vs EtOH**	U = 256	P = 0.8618
	**FD (EtOH)**	Mann-Whitney test	**Air vs EtOH**	U = 108	P < 0.0001
	**H**	Two-way ANOVA	**Air vs EtOH**	F (3, 88) = 5.433	P = 0.0018
	**I**	Two-way ANOVA	**(PF) Air vs (PF) EtOH**	F (3, 88) = 6.786	P = 0.0138
	**J**	Two-way ANOVA	**Air vs EtOH**	F (3, 88) = 9.313	P < 0.0001
**Sup Fig.1**	**C (Male, Presymptomatic, Body weight)**	Two-way ANOVA	**Air vs EtOH**	F (4, 62) = 0.3201	P = 0.8635
	**C (Female, Symptomatic, Body weight)**	Two-way ANOVA	**Air vs EtOH**	F (4, 100) = 0.8585	P = 0.4917
	**D (Male, Presymptomatic, Body weight)**	Two-way ANOVA	**Air vs EtOH**	F (3, 32) = 0.3373	P = 0.7984
	**D (Female, Symptomatic, Body weight)**	Two-way ANOVA	**Air vs EtOH**	F (3, 31) = 0.6150	P = 0.6105
**Sup Fig.3**	**A (ApoE, Cortex, Male)**	Mann-Whitney test	**Air vs EtOH**	U = 0	P = 0.0079
	**A (ApoE, Cortex, Female)**	Mann-Whitney test	**Air vs EtOH**	U = 1	P = 0.0159
	**A (ApoE, Hippocampus, Male)**	Mann-Whitney test	**Air vs EtOH**	U = 0	P = 0.0079
	**A (ApoE, Hippocampus, Female)**	Mann-Whitney test	**Air vs EtOH**	U = 2	P = 0.0317
	**B (GFAP, Cortex, Male)**	Mann-Whitney test	**Air vs EtOH**	U = 1	P = 0.0159
	**B (GFAP, Cortex, Female)**	Mann-Whitney test	**Air vs EtOH**	U = 0	P = 0.0079
	**B (GFAP, Hippocampus, Male)**	Mann-Whitney test	**Air vs EtOH**	U = 0	P = 0.0079
	**B (GFAP, Hippocampus, Female)**	Mann-Whitney test	**Air vs EtOH**	U = 0	P = 0.0079
	**C (ApoE, Cortex, Male)**	Mann-Whitney test	**Air vs EtOH**	U = 1.5	P = 0.0238
	**C (ApoE, Cortex, Female)**	Mann-Whitney test	**Air vs EtOH**	U = 0	P = 0.0079
	**C (ApoE, Hippocampus, Male)**	Mann-Whitney test	**Air vs EtOH**	U = 3	P = 0.0478
	**C (ApoE, Hippocampus, Female)**	Mann-Whitney test	**Air vs EtOH**	U = 3	P = 0.0456
**Sup Fig.4**	**A**	Mann-Whitney test	**Air vs EtOH**	U = 40.5	P = 0.4926
	**B**	Mann-Whitney test	**Air vs EtOH**	U = 37	P = 0.3423
	**C**	Mann-Whitney test	**Air vs EtOH**	U = 26	P = 0.0753
	**D**	Mann-Whitney test	**Air vs EtOH**	U = 9	P = 0.0011
	**E**	Mann-Whitney test	**Air vs EtOH**	U = 31	P = 0.1649
	**F**	Mann-Whitney test	**Air vs EtOH**	U = 47	P = 0.8534
	**G**	Mann-Whitney test	**Air vs EtOH**	U = 38	P = 0.3930
	**H**	Mann-Whitney test	**Air vs EtOH**	U = 10	P = 0.0278
**Sup Fig.5**	**A (HDL-C, Cortex, Male)**	Mann-Whitney test	**Air vs EtOH**	U = 7	P = 0.3095
	**A (HDL-C, Cortex, Female)**	Mann-Whitney test	**Air vs EtOH**	U = 8.5	P = 0.4603
	**A (HDL-C, Hippocampus, Male)**	Mann-Whitney test	**Air vs EtOH**	U = 12.5	P > 0.9999
	**A (HDL-C, Hippocampus, Female)**	Mann-Whitney test	**Air vs EtOH**	U = 7	P = 0.3095
	**B (LDL-C, Cortex, Male)**	Mann-Whitney test	**Air vs EtOH**	U = 1	P = 0.0159
	**B (LDL-C, Cortex, Female)**	Mann-Whitney test	**Air vs EtOH**	U = 1	P = 0.0159
	**B (LDL-C, Hippocampus, Male)**	Mann-Whitney test	**Air vs EtOH**	U = 2.5	P = 0.0397
	**B (LDL-C, Hippocampus, Female)**	Mann-Whitney test	**Air vs EtOH**	U = 2	P = 0.0317
	**C (Total-C, Cortex, Male)**	Mann-Whitney test	**Air vs EtOH**	U = 7	P = 0.3095
	**C (Total-C, Cortex, Female)**	Mann-Whitney test	**Air vs EtOH**	U = 12	P > 0.9999
	**C (Total-C, Hippocampus, Male)**	Mann-Whitney test	**Air vs EtOH**	U = 11	P = 0.8413
	**C (Total-C, Hippocampus, Female)**	Mann-Whitney test	**Air vs EtOH**	U = 9	P = 0.5476
**Sup Fig.6**	**A**	One-way ANOVA	**Air vs EtOH (Male)**	F (3, 9) = 1.031	P = 0.9636
			**Air vs EtOH (Female)**		P = 0.5892
	**B**	One-way ANOVA	**Air vs EtOH (Male)**	F (3, 9) = 0.9527	P = 0.7369
			**Air vs EtOH (Female)**		P = 0.5858
	**C**	One-way ANOVA	**Air vs EtOH (Male)**	F (3, 11) = 8.388	P = 0.0209
			**Air vs EtOH (Female)**		P = 0.4809
	**D**	One-way ANOVA	**Air vs EtOH (Male)**	F (3, 10) = 0.6277	P = 0.8978
			**Air vs EtOH (Female)**		P = 0.9988
	**E**	One-way ANOVA	**Air vs EtOH (Male)**	F (3, 11) = 12.16	P = 0.9998
			**Air vs EtOH (Female)**		P = 0.0015
	**F**	One-way ANOVA	**Air vs EtOH (Male)**	F (3, 10) = 1.222	P = 0.9984
			**Air vs EtOH (Female)**		P = 0.7469
	**G**	One-way ANOVA	**Air vs EtOH (Male)**	F (3, 12) = 0.7291	P = 0.9994
			**Air vs EtOH (Female)**		P = 0.0454
	**H**	One-way ANOVA	**Air vs EtOH (Male)**	F (3, 9) = 1.290	P = 0.9815
			**Air vs EtOH (Female)**		P = 0.4227
	**I**	One-way ANOVA	**Air vs EtOH (Male)**	F (3, 12) = 1.549	P = 0.2216
			**Air vs EtOH (Female)**		P = 0.9233
	**J**	One-way ANOVA	**Air vs EtOH (Male)**	F (3, 10) = 5.033	P = 0.0097
			**Air vs EtOH (Female)**		P = 0.9996
**Sup Fig.7**	**A**	Mann-Whitney test	**Air vs EtOH**	U = 12	P > 0.9999
	**B**	Mann-Whitney test	**Air vs EtOH**	U = 10	P = 0.6905
	**C**	Mann-Whitney test	**Air vs EtOH**	U = 7	P = 0.3095
	**D**	Mann-Whitney test	**Air vs EtOH**	U = 1	P = 0.0159
**Sup Fig.8**	**A (Aβ, Cortex, Male)**	Mann-Whitney test	**Air vs EtOH**	U = 2	P = 0.0317
	**A (Aβ, Cortex, Female)**	Mann-Whitney test	**Air vs EtOH**	U = 2	P = 0.0269
	**A (Aβ, Hippocampus, Male)**	Mann-Whitney test	**Air vs EtOH**	U = 0.5	P = 0.0159
	**A (Aβ, Hippocampus, Female)**	Mann-Whitney test	**Air vs EtOH**	U = 0	P = 0.0079
	**B (Aβ40, Cortex, Male)**	Mann-Whitney test	**Air vs EtOH**	U = 10	P = 0.2251
	**B (Aβ40, Cortex, Female)**	Mann-Whitney test	**Air vs EtOH**	U = 16	P = 0.7835
	**B (Aβ40, Hippocampus, Male)**	Mann-Whitney test	**Air vs EtOH**	U = 15.5	P = 0.7338
	**B (Aβ40, Hippocampus, Female)**	Mann-Whitney test	**Air vs EtOH**	U = 7.5	P = 0.1039
	**C (Aβ42, Cortex, Male)**	Mann-Whitney test	**Air vs EtOH**	U = 0	P = 0.0022
	**C (Aβ42, Cortex, Female)**	Mann-Whitney test	**Air vs EtOH**	U = 0	P = 0.0022
	**C (Aβ42, Hippocampus, Male)**	Mann-Whitney test	**Air vs EtOH**	U = 0	P = 0.0022
	**C (Aβ42, Hippocampus, Female)**	Mann-Whitney test	**Air vs EtOH**	U = 0	P = 0.0022
**Sup Fig.9**	**C**	Mann-Whitney test	**Air vs EtOH**	U = 38.5	P = 0.9136
	**D**	Mann-Whitney test	**Air vs EtOH**	U = 32	P = 0.4992
**Sup Fig.10**	**A (FDG, Cortex, Male)**	Mann-Whitney test	**Air vs EtOH**	U = 1.5	P = 0.0203
	**A (FDG, Cortex, Female)**	Mann-Whitney test	**Air vs EtOH**	U = 3	P = 0.0303
	**A (FDG, Hippocampus, Male)**	Mann-Whitney test	**Air vs EtOH**	U = 0	P = 0.0001
	**A (FDG, Hippocampus, Female)**	Mann-Whitney test	**Air vs EtOH**	U = 3	P = 0.0401
**Sup Fig.11**	**B**	Mann-Whitney test	**Air vs EtOH**	U = 8	P > 0.9999
	**C**	Mann-Whitney test	**Air vs EtOH**	U = 7	P = 0.8857
**Sup Fig.12**	**A**	Mann-Whitney test	**Air vs EtOH**	U = 101.5	P = 0.1414
	**B**	Mann-Whitney test	**Air vs EtOH**	U = 171.5	P = 0.8003
	**C**	Mann-Whitney test	**Air vs EtOH**	U = 110	P = 0.1626
	**D**	Mann-Whitney test	**Air vs EtOH**	U = 109	P = 0.1528
	**E**	Mann-Whitney test	**Air vs EtOH**	U = 299	P = 0.5061
	**F**	Mann-Whitney test	**Air vs EtOH**	U = 326	P = 0.7032
	**G**	Mann-Whitney test	**Air vs EtOH**	U = 62	P = 0.5899
	**H**	Mann-Whitney test	**Air vs EtOH**	U = 70	P = 0.9323
**Sup Fig.13**	**A**	Mann-Whitney test	**Air vs EtOH**	U = 122	P = 0.2112
	**B**	Mann-Whitney test	**Air vs EtOH**	U = 132	P = 0.3503
	**C**	Mann-Whitney test	**Air vs EtOH**	U = 23	P < 0.0001
	**D**	Mann-Whitney test	**Air vs EtOH**	U = 22	P < 0.0001
**Sup Fig.14**	**A**	Mann-Whitney test	**Air vs EtOH**	U = 26.5	P = 0.2457
	**B**	Mann-Whitney test	**Air vs EtOH**	U = 19.5	P = 0.0706
	**C**	Mann-Whitney test	**Air vs EtOH**	U = 20	P = 0.0831
	**D**	Mann-Whitney test	**Air vs EtOH**	U = 40	P > 0.9999
	**E**	Mann-Whitney test	**Air vs EtOH**	U = 28	P = 0.3154
	**F**	Mann-Whitney test	**Air vs EtOH**	U = 38	P = 0.8968
	**G**	Mann-Whitney test	**Air vs EtOH**	U = 26	P = 0.9546
	**H**	Mann-Whitney test	**Air vs EtOH**	U = 17	P = 0.1806
	**1**	Two-way ANOVA	**Air vs EtOH**	F (3, 40) = 1.476	P = 0.2356
	**J**	Two-way ANOVA	**Air vs EtOH**	F (3, 88) = 1.716	P = 0.1694
	**K**	Two-way ANOVA	**Air vs EtOH**	F (3, 40) = 1.204	P = 0.9961
**Sup Fig.15**	**A**	Mann-Whitney test	**Air vs EtOH (Male)**	U = 10.5	P = 0.0004
			**Air vs EtOH (Female)**	U = 56	P = 0.0113
	**B**	Mann-Whitney test	**Air vs EtOH (Male)**	U = 65.5	P = 0.9878
			**Air vs EtOH (Female)**	U = 118	P = 0.9766
	**C**	Mann-Whitney test	**Old vs New object (Air male)**	U = 45	P = 0.3653
			**Old vs New object (Air female)**	U = 47	P = 0.1600
	**D**	Mann-Whitney test	**Old vs New object (EtOH male)**	U = 15	P = 0.0019
			**Air vs Old vs New object (EtOH female)**	U = 42	P = 0.0027
	**E (Male)**	Two-way ANOVA	**Air vs EtOH**	F (3, 32) = 5.215	P = 0.0048
	**F (Male)**	Two-way ANOVA	**Air vs EtOH**	F (3, 48) = 2.693	P = 0.0465
	**G (Female)**	Two-way ANOVA	**Air vs EtOH**	F (3, 32) = 6.761	P = 0.0012
	**H (Female)**	Two-way ANOVA	**Air vs EtOH**	F (3, 88) = 3.638	P = 0.0191
**Sup Fig.16**	**A (ALT, Cortex)**	Mann-Whitney test	**Air vs EtOH**	U = 8	P = 0.1320
	**A (ALT, Hippocampus)**	Mann-Whitney test	**Air vs EtOH**	U = 14	P = 0.5887
	**A (ALT, Liver)**	Mann-Whitney test	**Air vs EtOH**	U = 0	P = 0.0022
	**B (AST, Cortex)**	Mann-Whitney test	**Air vs EtOH**	U = 16	P = 0.8182
	**B (AST, Hippocampus)**	Mann-Whitney test	**Air vs EtOH**	U = 8.5	P = 0.1450
	**B (AST, Liver)**	Mann-Whitney test	**Air vs EtOH**	U = 15	P = 0.6623
	**C (ALT/AST, Cortex)**	Mann-Whitney test	**Air vs EtOH**	U = 12	P = 0.3939
	**C (ALT/AST, Hippocampus)**	Mann-Whitney test	**Air vs EtOH**	U = 11	P = 0.3095
	**C (ALT/AST, Liver)**	Mann-Whitney test	**Air vs EtOH**	U = 0	P = 0.0022

## Data Availability

All data generated or analyzed during this study are included in this published article and its additional files.

## Abbreviations

AD: Alzheimer’s disease
Apo E: Apolipoprotein E
APP/PS1: Amyloid precursor protein and presenilin 1
ARD: Alcohol-related dementia
AUD: Alcohol use disorder
Aβ: Amyloid beta
ELISA: Enzyme Linked Immunosorbent Assay
FDG-PET: Fludeoxyglucose-positron emission tomography
GFAP: Glial fibrillary acidic protein
HMG-CoA: 3-hydroxy-3-methyl glutaryl-CoA
IHC: Immunohistochemistry
HOC: Inhibitor of nuclear factor-κB kinase
IL-1β: interleukin 1beta
LDL: Low-density lipoprotein
LRP1: Low-density lipoprotein receptor-related protein 1
MEE: Moderate ethanol exposure
MEE: Moderate ethanol exposure
NF-κB: Nuclear factor kappa B
NOR: Noble objective recognition
PD: Parkinson’s disease
TLR4: Toll-like receptor 4
TNF-α: Tumor Necrosis Factor Alpha
